# Analysing the Zenith Tropospheric Delay Estimates in On-line Precise Point Positioning (PPP) Services and PPP Software Packages

**DOI:** 10.3390/s18020580

**Published:** 2018-02-14

**Authors:** Jorge Mendez Astudillo, Lawrence Lau, Yu-Ting Tang, Terry Moore

**Affiliations:** 1 International Doctoral Innovation Centre, University of Nottingham Ningbo China, Ningbo 315100, China; Jorge.mendez@nottingham.edu.cn; 2 Department of Civil Engineering, University of Nottingham Ningbo China, Ningbo 315100, China; 3 School of Geographical Sciences, University of Nottingham Ningbo China, Ningbo 315100, China; Yu-Ting.Tang@nottingham.edu.cn; 4 The Nottingham Geospatial Institute, University of Nottingham, Nottingham NG7 2RD, UK; Terry.Moore@nottingham.ac.uk

**Keywords:** GNSS, Zenith Tropospheric Delay, Precise Point Positioning, GNSS meteorology

## Abstract

As Global Navigation Satellite System (GNSS) signals travel through the troposphere, a tropospheric delay occurs due to a change in the refractive index of the medium. The Precise Point Positioning (PPP) technique can achieve centimeter/millimeter positioning accuracy with only one GNSS receiver. The Zenith Tropospheric Delay (ZTD) is estimated alongside with the position unknowns in PPP. Estimated ZTD can be very useful for meteorological applications, an example is the estimation of water vapor content in the atmosphere from the estimated ZTD. PPP is implemented with different algorithms and models in online services and software packages. In this study, a performance assessment with analysis of ZTD estimates from three PPP online services and three software packages is presented. The main contribution of this paper is to show the accuracy of ZTD estimation achievable in PPP. The analysis also provides the GNSS users and researchers the insight of the processing algorithm dependence and impact on PPP ZTD estimation. Observation data of eight whole days from a total of nine International GNSS Service (IGS) tracking stations spread in the northern hemisphere, the equatorial region and the southern hemisphere is used in this analysis. The PPP ZTD estimates are compared with the ZTD obtained from the IGS tropospheric product of the same days. The estimates of two of the three online PPP services show good agreement (<1 cm) with the IGS ZTD values at the northern and southern hemisphere stations. The results also show that the online PPP services perform better than the selected PPP software packages at all stations.

## 1. Introduction

GNSS data is widely used for positioning and navigation in mass-market and engineering applications [1] and for altitude determination [2], moreover, it can also be used for monitoring the atmosphere. The electron content in the ionosphere and the air density in the electrically neutral atmosphere (troposphere) affect GNSS signals propagating through the atmosphere. The influence of the troposphere is described by the total refractivity N, which depends on pressure, temperature and water vapor partial pressure [3]. An example of the use of GNSS data for applications other than positioning and navigation is the integration of the GNSS-derived Path Delay with microwave radiometer measurements to find a precise wet tropospheric correction for altimetric products [4]. Another use of GNSS data is the remote sensing of the atmosphere, where GNSS signals can be used to measure physical variables such as atmospheric temperature, pressure and tropopause heights needed for weather and climate change monitoring [5]. Other examples are the measurement of the amount of precipitable water in the atmosphere using GPS signals as in the experiment GPS/MET [6,7]. The amount of precipitable water can be estimated from the amount of water vapor in the atmosphere which is proportional to the Zenith Wet Delay (ZWD) [8,9,10], which is relevant to weather forecasting and the study of extreme weather phenomena. Moreover, three-dimensional water vapor can be reconstructed from GNSS observations from different systems [11].

The ZWD and the Zenith Hydrostatic Delay (ZHD) comprise the total Zenith Tropospheric Delay (ZTD) that can be estimated with the Precise Point Positioning (PPP) technique. PPP estimates the tropospheric delay, the position and clock offsets using precise ephemeris and the ionospheric-free combinations of dual-frequency GPS pseudorange and carrier-phase observations [12].

Given the increasing attention of PPP among the GNSS community, different software packages have been developed. The precision of the positioning results can be assessed, for example, the online PPP solution by the University Of New Brunswick (GAPS) has been evaluated in terms of its achievable accuracy [13]. An overview of GNSS data analysis capabilities that can be implemented in PPP software GAPS has been investigated [14]. Moreover, the analysis of the accuracy of the position determination using single-receiver GNSS measurements with different observing conditions using the online software CSRS-PPP has been investigated [15]. In terms of the use of GNSS data for meteorology, the results of the Integrated Water Vapor (IWV) estimated from GPS and Galileo observables have been compared with the results obtained with a radiosonde [16] and it was concluded that the Galileo-GPS IWV estimates are close to those of GPS-only at a level of 0.13 kg/m^2^ of precipitable water.

Studies about estimated ZTD with PPP are scarce, the results of estimated ZTD with the Automatic Precise Positioning Service (APPS), the GPS Analysis and Positioning Software, Canadian Spatial Reference System precise point positioning service (CSRS-PPP), and the Magic-PPP online PPP software have been compared to assess the quality of positioning estimation [17]. The suitability of real-time ZTD estimates obtained from three different PPP software packages, the PPP wizard, developed by the University of Luxembourg, the Tefnut application developed by the Geodetic Observatory Pecny and the BKG Ntrip Client developed by the Bundesamt fuer Kartografie und Geodaesie, (all of them capable of performing PPP in real time) has been assessed by comparing them with the IGS final troposphere product as well as with collocated radiosonde observations. The motivation for such assessment was to find which precise ZTD estimates can be used in Numeric Weather Prediction models [18].

However, there has been no assessment on the quality of ZTD-estimates using data from stations in different latitudes and in the four seasons using both, online and post-processing PPP software packages. Also, the quality of ZTD-estimates obtained with the software developed by the University of Nottingham (POINT) has not yet been assessed.

This paper presents a comparative analysis of ZTD-estimates obtained with six different software packages using eight days of data from nine IGS stations. The ZTD estimated with JPL’s APPS, CSRS-PPP, MagicGNSS, European Space Agency and Barcelona’s tech GNSSLab Tool (gLAB), the open source RTKLIB and the University of Nottingham’s POINT using eight days (two days during winter, two days during spring, two days during summer and two days during autumn) of observation data from nine IGS stations around the world are compared with the ZTD-estimates obtained from the IGS tropospheric product of the same day epoch by epoch in order to assess the quality of the estimation by comparing which estimated ZTD is closer to the value provided by the IGS Tropospheric Product using the Root Square Mean Error of the differences. The results of the comparison are grouped depending on the station’s latitude in order to investigate if the quality of the estimates depend on the latitude of the station, this analysis helps users to select the most appropriate PPP software for regional ZTD determination in the user latitude. Then, the results are grouped by season of the year when the data was collected, in order to evaluate the effect of the weather. Finally, all the results obtained with the same software are grouped in order to compare the quality of estimations with each of the software packages. The days chosen for this assessment are days 027,118,208 and 300 of year 2016 and days 027,117,207 and 299 of year 2017, covering different meteorological conditions during different seasons. This assessment is relevant for GNSS meteorology applications such as water vapor calculation using ZWD and Numeric Weather Prediction models where a precise tropospheric delay is needed.

### 1.1. Tropospheric Delay

GNSS signals propagating through the atmosphere are delayed due to the free electron content in the ionosphere and by the air density in the electrically neutral atmosphere also called troposphere. The refractive index *n* or the total refractivity *N* of the troposphere is described by:

(1) $$N=10^{6}\left(n-1\right)$$

The total refractivity of the troposphere can be separated into two main components, the hydrostatic or dry ($N_{dry}$) and the wet component ($N_{wet}$) caused by dry gases and the water vapor respectively [19] and it can be expressed as a function of meteorological parameters such as air pressure *p*, temperature *T* and water vapor partial pressure *e* [3]:

(2) $$N=N_{dry}+N_{wet}=k_{1}\cdot \frac{p-e}{T}+k_{2}\cdot \frac{e}{T}+k_{3}\cdot \frac{e}{T^{2}}$$

where *k*_1_ = 77.689 K·h·Pa^−1^, *k*_2_ = 71.295 K·h·Pa^−1^ and *k*_3_ = 375,463 K^2^·h·Pa^−1^ are empirically determined coefficients [20]. The troposphere causes a delay to the signal $\Delta^{PD}$ which can be expressed as an integral of the total refractivity *N* along the propagation path *s* from receiver *r* to the satellite *w*:

(3) $$\Delta^{PD}=10^{-6}\int_{r}^{w}Nds$$

The tropospheric delay can also be separated in the hydrostatic and the wet component. Therefore, Equation (3) can be written as:

(4) $$\Delta^{PD}=10^{-6}\int_{r}^{w}N_{dry}ds+10^{-6}\int_{r}^{w}N_{wet}ds$$

The total tropospheric delay in slant path delay can be mapped to the zenith direction, yielding the Zenith Tropospheric Delay (ZTD) using a mapping function depending on the elevation angle of the satellite. The ZTD is defined as the addition of the Zenith Hydrostatic Delay (ZHD) and the Zenith Wet Delay (ZHD) which are the left and the right side of Equation (4):

(5) ZTD=ZHD+ZWD

(6) $$\Delta^{PD}=ZHD*m_{h}\left(E\right)+ZWD*m_{w}\left(E\right)$$

where $m_{h}\left(E\right)$ and $m_{w}\left(E\right)$ are the hydrostatic and wet mapping functions depending on the elevation angle. The ZTD can be determined as an integral of *N* in the zenith direction [20]:

(7) $$ZTD=10^{-6}\int_{zenith\;direction}^{}Nds$$

Equation (7) indicates that the Zenith Tropospheric Delay and the refractivity of the troposphere are related. Since the refractivity depends on meteorological conditions along the signal path, the ZTD can be related to these conditions. The ZTD is a parameter estimated in the Precise Point Positioning technique.

### 1.2. Precise Point Positioning

Undifferenced pseudorange and carrier phase observations from a single GPS receiver are processed in the PPP technique [21]. The technique is named “precise” because precise *a priori* information such as satellites orbits and clock errors from different sources like the International GNSS Service (IGS) [22], the Jet Propulsion Lab (JPL) or Natural Resources Canada (NRCan) [23] are used in the data processing. The advantages of this technique are the accuracies obtained (cm-level with only one receiver [23]) and that it eliminates the need to acquire simultaneous tracking data from a reference station or from a network of stations [22]. Moreover, the use of a single receiver reduces equipment cost and makes the processing less labor and resources intensive.

### 1.3. Observation Equations

The ionosphere causes a delay to the GNSS signal propagating through the atmosphere which can be reduced greatly with dual-frequency data. The ionosphere-free combinations of dual-frequency GPS pseudoranges (P) and carrier-phase observations (ϕ) are related to user position, clock, troposphere and ambiguity parameters according to the following simplified observation equations [12]:

(8) $$l_{p}=\rho +C\left(dt-dT\right)+Tr+\varepsilon_{P}$$

(9) $$l_{\varphi }=\rho +C\left(dt-dT\right)+Tr+B\lambda +\varepsilon_{\varphi }$$

where $l_{p}$ is the ionosphere-free combination of L1 and L2 pseudoranges, $l_{\varphi }$ is the ionosphere-free combination of L1 and L2 carrier-phases, *dt* is the receiver’s clock offset from GPS time, *dT* is the satellite clock offset from GPS time, C is the speed of light in vacuum, *Tr* is the signal path delay due to the neutral-atmosphere (primarily the troposphere), *λ* is the carrier wavelength, *B* is the ambiguity of the carrier-phase ionosphere-free combination, and $\varepsilon_{p}$ and $\varepsilon_{\varphi }$ are the measurement noise components. ρ is the geometrical range computed as a function of satellite (*Xs*,*Ys*,*Zs*) and station coordinates (*x*,*y*,*z*) defined as:

(10) $$\rho =\sqrt{{\left(Xs-x\right)}^{2}+{\left(Ys-y\right)}^{2}+{\left(Zs-z\right)}^{2}}$$

the position, the tropospheric delay, the ambiguity and the clock offsets need to be estimated using estimators such as the Extended Kalman Filter (EKF) or the Least Squares technique.

## 2. Materials and Methods

The PPP technique has been implemented by different software packages which follow different estimation strategies or use precise ephemeris from different sources. There are online services accessible through the Internet and software packages that have to be installed and run locally in a computer. For this study, six PPP post-processing software packages were used in total, three of them are online services, APPS, CSRS-PPP and MagicGNSS and three stand-alone post-processing software packages gLAB, POINT and RTKLIB. A summary of the characteristics of the online-services and the software used for this study can be found in Table 1.

The Automatic Precise Positioning Service (APPS) is an online service by the Jet Propulsion Lab which can estimate position coordinates as a single set in Static Mode or a time series in Kinematic Mode. In APPS the receiver clock states are estimated as white noise with updates every measurement epoch and the Zenith Wet delay (ZWD) is estimated as a random walk with variance of 3 ${\mathrm{mm}}^{2}$ per hour. Moreover, the wet delay gradient is estimated as a random walk with variance of 0.3 ${\mathrm{mm}}^{2}$ per hour and the phase ambiguities are estimated as real numbers [24]. 

APPS can take only dual-frequency GPS observations. The service allows the user to decide whether to use Final, Rapid and Ultra-rapid type products from the Jet Propulsion Lab (JPL) for corrections of obits and clocks of satellites. The ZTD is estimated applying the Global Mapping Function troposphere mapping function with an *a priori* hydrostatic delay of $1.013*2.27*e^{-0.000116*h}$ meters where *h* is the station height above the ellipsoid in meters and *a priori* wet delay of 0.1 m. The wet delay is estimated together with positioning unknowns.

The Canadian Spatial Reference System (CSRS-PPP service) by Natural Resources Canada uses a dynamic filter to estimate the station position in static or kinematic mode, the station-clock states, the local tropospheric zenith delays and the carrier-phase ambiguities. The approach used in CSRS-PPP for ZTD estimation is to smooth the estimates by a backward substitution with the final converged satellite ambiguity parameters held fixed for all epochs.

This approach is implemented to obtain optimal station Zenith Path Delay time series based on all observations within the observation session [12]. Precise corrections to orbits and clocks of satellites used are made available by the IGS. The mapping function used in CSRS-PPP is the Global Mapping function. As an input, single or dual-frequency GNSS data can be used. The user may choose NAD83 or ITRF2008 frame of reference to determine coordinates [12].

The service of MagicGNSS operated by the company GMV Aerospace and Defense is made available through their website where the user can process data in static and kinematic mode at two frequencies. The user can choose to use final and rapid products for corrections of orbits and clocks of satellites made accessible by the IGS or GMV, the user can choose either rapid or final products. The current version can process data from constellations, GPS, GLONASS, Galileo, BeiDou. Coordinates of the calculated position can be determined in two frames of reference ITRF2008 or ETRS89. MagicGNSS does not take into account in calculation parameters of the phase center antenna.

The following three software packages had to be installed in the computer and had to be run locally in the processing computer. For all of them, it is necessary to load the observation file, the navigation file and for PPP, the precise ephemeris are also needed. Additionally, other corrections such as Ocean Tide Loading or the parameters of the phase center antenna can be included. Since the user has to load all the files, there is flexibility to use corrections from different sources. For this study, all the final products from the IGS were used.

gLAB is an advanced interactive educational multipurpose package to process and analyze GNSS data [25] developed by Catalonia Technical University and the European Space Agency. It can process either single or dual-frequency GPS only data. The tropospheric delay is defined in terms of the elevation angle (El) of the satellite as:

(11) $$T_{r}\left(El\right)=T_{rz,dry}M_{dry}\left(El\right)+T_{rz,wet}M_{wet}\left(El\right)$$

where $T_{rz,dry}$ and $T_{rz,wet}$ are the dry and wet slant tropospheric delay which can be estimated with a simple model:

(12) $$T_{rz,dry}=\propto e^{\;\beta H}$$

(13) $$T_{rz,wet}=T_{rz0,wet}+\Delta T_{rz,wet}$$

where $\propto =2.3m,\;\beta =0.116\cdot 10^{-3}$ *H* is the height above sea level, in meters. $T_{rz0,wet}=0.1\;m$ and $\Delta T_{rz,wet}$ is estimated as a random walk process in the navigation-Kalman-filter together with the coordinates and other parameters [25,26,27]. Or with the UNB-3 model.

*M_dry_* and *M_wet_* are the dry and wet mapping function (Neill mapping function) which does not require any meteorological data. The multiplication of the mapping function and the slant delay yield the Zenith Troposheric Delay.

POINT is a software package developed by the University of Nottingham. It is capable of processing L1 and L2 GPS data. It implements an Extended Kalman Filter for positioning employing double differences observables [28]. The hydrostatic component of the ZTD is calculated using a model such as Saastamoinen, Hopfield or IFADIS and the Neill mapping function. The wet component is an unknown in the Extended Kalman Filter, the total zenith tropospheric delay is calculated as the sum of wet and dry components:

(14) $$ZTD=ZHD_{model}+ZWD_{estimated}$$

RTKLIB is an open source positioning software developed by Takatsu. It can implement different positioning techniques, among them, PPP which can be computed in static or kinematic mode. All the corrections are input to the software via its Graphic User Interface. The effect of the troposphere is modelled using a mapping function and zenith tropospheric delays. The mapping function in terms of the elevation angle (El) and the azimuth angle (Az) between the satellite and the receiver is calculated as:

(15) $$M\left(El\right)=M_{w}\left(El\right)\left\{1+\cot\left(El\right)\left(G_{N}\cos\left(Az\right)+G_{E}\sin\left(Az\right)\right)\right\}$$

and the tropospheric delay is calculated as:

(16) $$T_{r,z}=M_{h}\left(El\right)Z_{H}+M\left(El\right)\left(Z_{T}-Z_{H}\right)$$

where $Z_{T}$ is the tropospheric zenith total delay in meters. This parameter is estimated from the Extended Kalman Filter together with the north component of tropospheric gradient ($G_{N,}$) and the east component of tropospheric gradient ($G_{E,}$). $Z_{H}$ is the tropospheric zenith hydro-static delay in meters which is calculated using a tropospheric model, either Saastamoinen, Hopfield or modified Hopfield model with the zenith angle *z* = 0 and relative humidity *hrel* = 0. $M_{h}\left(El\right)$ and $M_{w}\left(El\right)$ are the hydro-static and wet mapping function respectively. The Niell Mapping Function (NMF) is used in both cases. A summary of the capabilities of the software used for this study is shown in Table 1.

The three PPP software used for this experiment were: gLAB, POINT and RTKLIB which require precise ephemeris and other corrections to be input manually. In all cases, the corresponding IGS final clock and ephemeris files were used which contain data every 15 min. The three PPP software required the ANTEX file for antenna phase correction. No decimation was chosen for neither of the software, therefore, a solution was found for every epoch available in the observation file and the ephemeris file. The elevation mask was set to 10 degrees as indicated in Table 1 and GPS-only data was used in all software. The UNB-3 Nominal model was used together with the Neil Mapping function to model the effect of the troposphere in POINT and gLAB.

POINT requires more files to process data using the PPP technique. The differential code bias product (DCB) computed by the Institute of Geodesy and Geophysics of the Chinese Academy of Sciences was used. The Ocean Tide Loading (OTL) file obtained with the GOT00.2 model was also included and the same ANTEX file as for gLAB was used as well. The Saastamoinen model is used to compute the hydrostatic component of the tropospheric delay and the wet component is estimated as an unknown in the Extended Kalman Filter. RTKLIB had as input the same ephemeris file (Final from the IGS) and the same corrections as POINT, the same DCB, OTL and ANTEX files were used, it was run in PPP kinematic mode, the elevation mask was set to 10 degrees. The tropospheric effect was calculated as an unknown in the Extended Kalman Filter.

The data used in this experiment was collected from the IGS which operates over 400 GNSS stations across the world, it provides daily and hourly observation and navigation files for each station. Furthermore, the IGS provides other products, such as satellite ephemeris, earth rotation parameters and tropospheric delay with different latencies. The tropospheric delay product is generated from ground-based GNSS data with the Bernese GPS Software version 5.0, a cut-off angle of 7, IGS final satellite, orbit and EOP products are used for the computation [29]. Therefore, the IGS ZTD product is available approximately after three weeks after the observation date once the final products are available. The product contains the estimation of clock, position of the receiver which is presented as a constant, zenith delay in millimeters, which is estimated as a random walk with variance of 3 cm/h. Also included are the atmospheric gradients estimated as a random walk with variance of 0.3 cm/h. The temporal resolution of zenith day estimates is 5 min and the mapping function is the Global Mapping Function (GMF). For this study, observation data from 9 IGS stations listed in Table 2 were used as well as the IGS tropospheric product for 8 days. The days chosen for the study were the 27th calendar day of the month of January, April, July and October of the year 2016 and 2017 because these dates cover weather conditions during the four seasons in the different hemispheres.

## 3. Results

**Figure 1:**
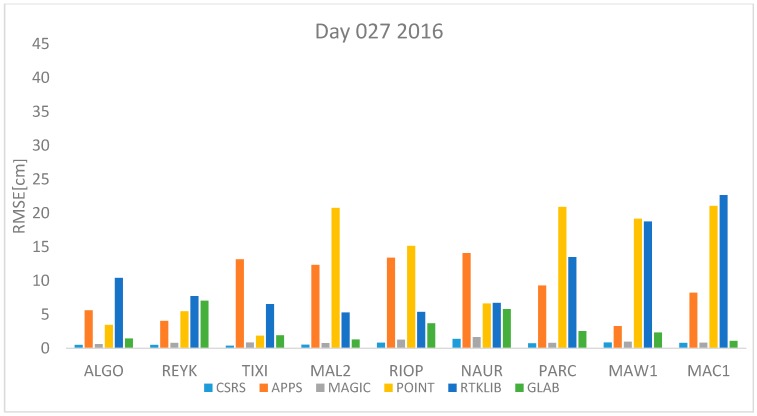
RMSE in centimeters for day 27 2016.

**Figure 2:**
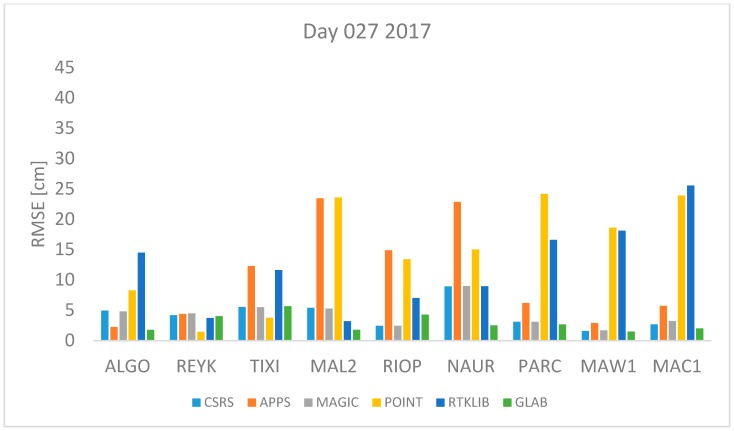
RMSE in centimeters for day 27 2017.

**Figure 3:**
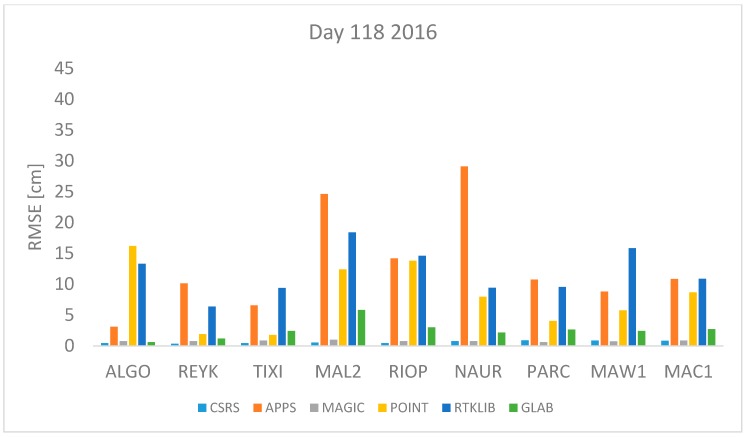
RMSE in centimeters for day 118 2016.

**Figure 4:**
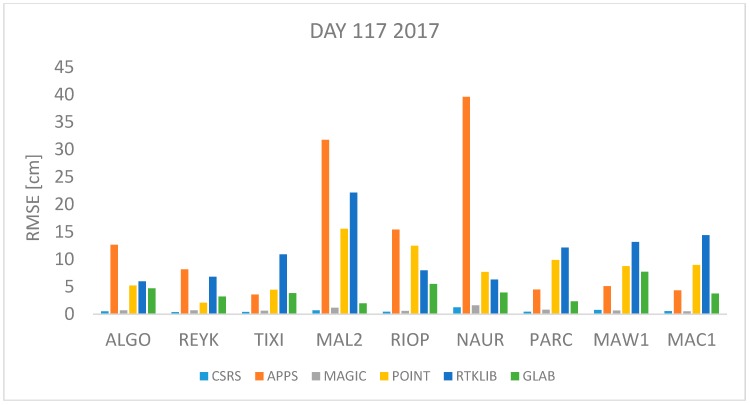
RMSE in centimeters for day 117 2017.

**Figure 5:**
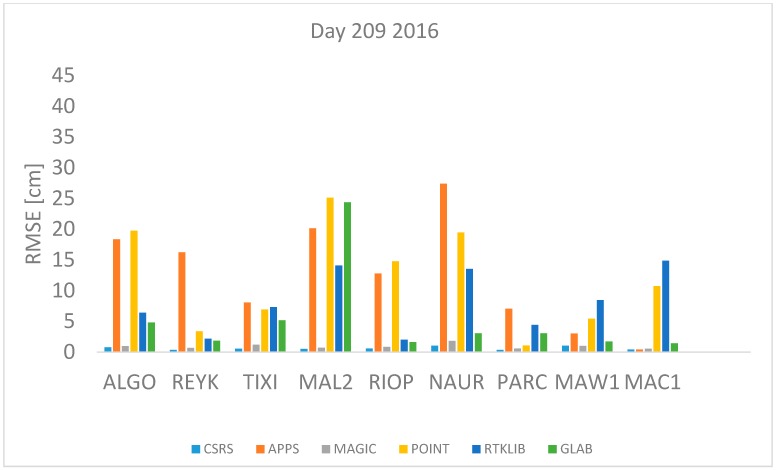
RMSE in centimeters for day 209 2016.

**Figure 6:**
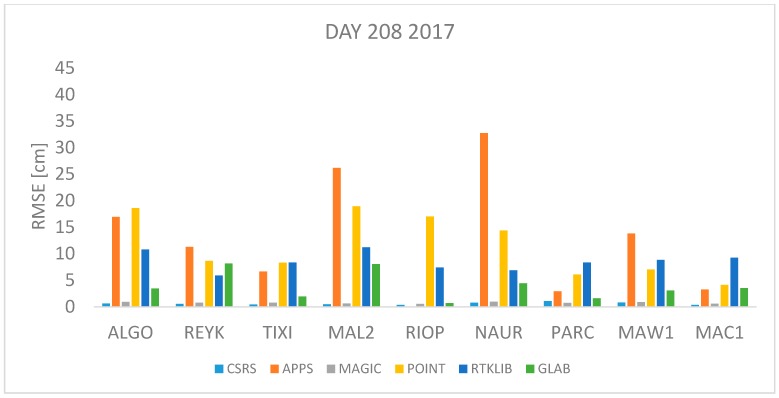
RMSE in centimeters for day 208 2016.

**Figure 7:**
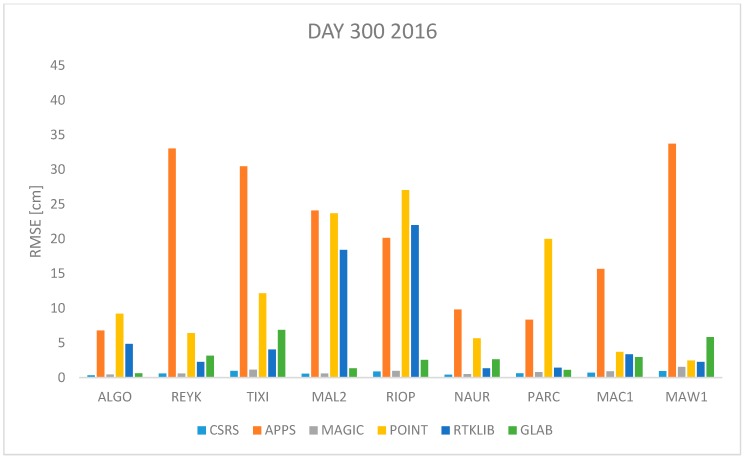
RMSE in centimeters for day 300 in year 2016.

**Figure 8:**
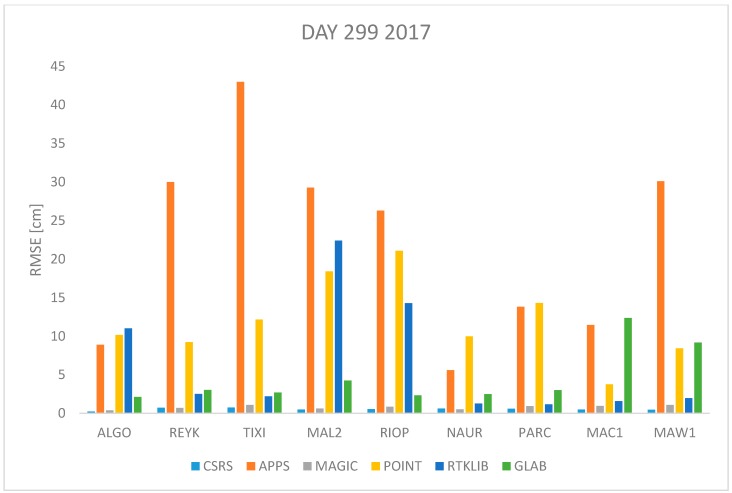
RMSE in centimeters for day 299 in year 2017.

In order to assess the quality of the ZTD estimates of the 6 PPP software previously described, observation data from the 9 IGS stations were processed with each of the software packages and the ZTD was estimated in kinematic mode. The Root Mean Square Error (RMSE) is used as the quality indicator in this performance assessment, which is computed as:

(17) $$RMSE=\sqrt{\frac{\sum_{i=1}^{n}{\left(ZTD_{estimated}-ZTD_{IGS}\right)}^{2}}{n}}$$

where *n* is the total number of *ZTD* estimates available. The *RMSE* was computed for each station and each software per day using Equation (17). All estimates available were used. The results are shown in Figure 1, Figure 2, Figure 3, Figure 4, Figure 5, Figure 6, Figure 7 and Figure 8. The first three stations are in the northern hemisphere, the next three are near the equator and the last three are in the southern hemisphere, the data was grouped according to the station location and the RMSE of the differences for each group was calculated and the results are shown in Table 3, Table 4, Table 5 and Table 6. Finally, Table 7 shows the RMSE of all differences estimated with each software and each online PPP service.

Figure 1 and Figure 2 represent the 27th of January of 2016 and 2017 respectively. This is the winter in the northern hemisphere and the summer in the southern hemisphere. In both figures, it can be seen that the estimates obtained with APPS and POINT for the stations MAL2 and NAUR are in both cases very far away from the value from the IGS tropospheric product. Also, station MAL2 and RIOP produce very high values of RMSE with POINT and APPS. However, in both days, the RMSE obtained with CSRS-PPP and MagicGNSS are lower than 5 cm for all stations. Furthermore, high RMSE values for the stations PARC, MAW1 and MAC1 are obtained with RTKLIB and POINT.

April 27th 2016 and 2017 are days 118 2016 and 117 2017 respectively. This is a day in spring in the northern hemisphere or autumn in the southern hemisphere. Therefore, mild changes of temperature are expected. The RMSE values of the differences between the estimated ZTD and the IGS tropospheric product’s value are depicted in Figure 3 and Figure 4 from where it can be seen that APPS, POINT and RTKLIB obtain a high RMSE value for the station MAL2 and NAUR. In contrast, the RMSE value is very low in all stations (<5 cm) for the estimates obtained with CSRS-PPP and MagicGNSS.

Day 209 2016 and 208 2017 correspond to 27 July 2016 and 2017 respectively which is a day in summer in the northern hemisphere with high temperatures expected or winter in the southern hemisphere with low temperatures expected. Figure 5 and Figure 6 depict the RMSE values for day 209 and 208 respectively, it can be seen that the highest RMSE values are found with data from stations NAUR, MAL2 and RIOP processed with APPS, POINT and RTKLIB. In contrast, the lowest RMSE values are found with CSRS-PPP and MagicGNSS for all stations.

Figure 7 and Figure 8 show the RMSE for data from 26 October 2016 and 2017 (day 300 and 299 respectively). This is a day in autumn in the northern hemisphere, cold temperatures expected, or spring, mild temperatures expected, in the southern hemisphere. According to data presented in Figure 7 and Figure 8 the RMSE obtained with APPS very high (more than 20 cm) for stations REYK, TIXI and MAW1 while the RMSE obtained with CSRS-PPP and MagicGNSS remains low under two centimeters for all cases.

Figure 1, Figure 2, Figure 3, Figure 4, Figure 5 and Figure 6 show a trend that in most cases the RMSE obtained with data from stations near the equator is higher than for the other stations with most of the software used for implementing the PPP technique. In order to further study the quality of ZTD estimation at different latitudes, the stations were grouped in three groups according on their latitude and the GNSS data from all the stations in the same latitude was compared to the respective IGS Tropospheric Product by calculating the difference between both values. The RMSE of all the differences from the stations in the group was calculated. These results are presented in Table 3, Table 4, Table 5 and Table 6. The regions are defined as: North: ALGO, REYK and TIXI, Center: MAL2, RIOP and NAUR and South: PARC, MAW1 and MAC1.

According to the results shown in Table 3, Table 4, Table 5 and Table 6 CSRS-PPP is the online software that performs the best for all stations because the RMSE is always lower than RMSE from other software. According to the same data, most of the software used for this study had the highest RMSE value with data from the equatorial stations. APPS, POINT and RTKLIB had their highest value of RMSE for the equatorial stations. MAGIC had most of its highest RMSE values in stations in the equatorial region except with data from July 2017, October 2016 and 2017. Similarly, CSRS had the highest RMSE for stations near the equator for five days, three days the highest RMSE was found for stations in the southern hemisphere. In contrast, GLAB did not have a clear pattern, three days the highest RMSE was found for the stations in the North, two days for the equatorial region and three days for the stations in the southern hemisphere.

In order to evaluate the quality of ZTD estimates of each software, the RMSE of all the differences (ZTD estimated from all stations with the same software minus IGS tropospheric product) was calculated. Its results are shown in Table 7 with all values in centimeters.

Table 7 shows that the two online services CSRS-PPP and MAGIC estimate the ZTD to a closer value to the IGS tropospheric product than the three other software packages for most cases the RMSE is equal or less than 1 cm (not the case with data from Day 27 of year 2017). From the three PPP software run locally GLAB is the one that had the lowest RMSE.

## 4. Discussion

Every software used for the analysis presented in this study uses a similar strategy to estimate the Zenith Tropospheric Delay, which is using a model to estimate the hydrostatic slant delay, use a mapping function to estimate the delay in the zenith direction and estimate the wet delay as an unknown in the parameter estimation process typically done with an Extended Kalman Filter. The online PPP software use the Global Mapping Function based on numerical weather model data while the locally run PPP processing software implement the Niell Mapping Function which depends only on the site coordinates and day of the year. Because the GMF involves the use of weather model data, it models better the delay caused by the troposphere which as seen in Equation (2) is affected by the meteorological variables near the receiver such as temperature, pressure, and partial water vapor pressure. The use of the GMF is one of the reasons why online services obtain an estimation closer to the IGS tropospheric product.

The effect of the ionosphere is another reason why the estimated value and the value of ZTD from the IGS tropospheric product are different. The model used to correct the ionosphere effect used by the online PPP services is not stated, for the other three locally-run software no ionosphere model is used, only the carrier phase and code combinations is done to obtain ionosphere-free pseudoranges, this combination only eliminates the first order ionosphere effect but residual effects are not eliminated and they can cause an effect on to the signal.

A third reason for the discrepancies between estimated ZTD and the values in the IGS tropospheric product is the different cut-off angle set up in the software. The IGS tropospheric product has a cut-off angle of 7. All the software packages and online PPP services used for this study allow to set the cut-off angle. However, the RTKLIB version used for this study allows to use only angles multiple of 5, therefore, 10 was used as the closest option. Also, in the other software the cut-off angle was set to 10 degrees. It is possible that this 3 degrees difference has an effect on the ZTD estimation because some satellites might be discarded for the solution. Furthermore, multipath affects the signal as well.

According to the results presented in this study, POINT and RTKLIB had very high RMSE values for stations near the equator which means that the model currently used does not clearly represent the tropospheric effect at these latitudes, possible reasons is that the thickness of the troposphere on the equatorial region is higher than in the polar region and different weather conditions near the equator. Also, APPS and MAGIC obtained the highest RMSE values for stations in the center for days 27 2016 and 2017, 118 2016, 117 2017 and 209 2016, however for the other days the highest values were found for the southern and northern hemisphere respectively which confirms that the equatorial region has specific atmospheric conditions that are not properly accounted for with models and the parameters used for the estimation. CSRS-PPP and GLAB obtained high RMSE values with data from different latitudes at different days. However, the results with CSRS-PPP were always less than 1 cm (except for day 27 2017) and in the case of GLAB the results were in the range of 2 and 6 cm always which means that both software obtained estimates very close to the IGS tropospheric value with all data. The solution with GLAB takes one epoch to converge, so the first epoch is not considered in the RMSE analysis.

This study only included eight days of data, however the same days in different years were chosen, also, the stations chosen are distributed throughout the globe. It is expected to find similar weather conditions the same days of two different years, therefore a trend can be found of how close the estimations are to the IGS tropospheric product. The stations are located in different latitudes which allows to study how the different models used for the tropospheric model are influenced by the latitude of the station.

## 5. Conclusions

In this paper, a comparison analysis of the estimated Zenith Tropospheric Delay (ZTD) obtained with 6 Precise Point Positioning (PPP) post-processing software and the International GNSS Service (IGS) tropospheric product is presented. The estimated ZTD obtained with APPS, CSRS-PPP, MagicGNSS, POINT, RTKLIB and gLAB were compared with the ZTD provided by IGS. The Root Mean Square Error (RMSE) was used as the indicator of accuracy of the estimation because it indicates how different the estimated value is from the ground truth.

Three trends were found in this study, first, it was found that CSRS-PPP obtains ZTD estimates very close to the value from the IGS Tropospheric product. Second, it was found that the tropospheric models currently implemented in RTKLIB and POINT do not account properly to the weather and atmospheric conditions in the equatorial region. The corrections used by CSRS-PPP and MAGIC are very precise so estimates closer to the truth value were found. The third trend found was that GLAB also estimates the ZTD to a value very close to the IGS Troposheric product.

The season change did not have a big impact on the ZTD estimation by PPP software. With the selected data sets. If precise ZTD estimates are needed for GNSS meteorology or numeric weather models, CSRS-PPP can provide very accurate estimates followed by MagicGNSS and gLAB.

## Figures and Tables

**Table 1:** Comparison of capabilities of the software packages used in this study.

Parameter	APPS	CSRS-PPP	magicGNSS	gLAB	POINT	RTKLIB
Version	GIPSY 6.4	1.05	N/A	5.0.0	N/A	2.4.3
Mode of calculation	Static/kine-matic	Static/kine-matic	Static/kine-matic	Static/kine-matic	Static/kine-matic	Static/kine-matic
Constellation	GPS	GPS,GLO	GPS,GLO, Galileo,BDS	GPS, GLO,Galileo	GPS,GLO	GPS,GLO, GPS+GLO
Frequency	L1,L2	L1,L2	L1,L2	L1,L2	L1,L2	L1,L2
Type of observation	Code and phase	Code and phase	Code and phase	Code and phase	Code and phase	Code and phase
Antenna model	Not taken into account	Taken into account	Not taken into account	Taken into account	Taken into account	Taken into account
Frame of reference	ITRF2008	ITRF2008	ITRF2008	ITRF2008	ITRF2008	ITRF2008
Orbits and clocks of satellites	JPL final	IGS final	GMV Rapid, IGS Rapid, IGS final	IGS final	IGS final	IGS final
Cut-off angle	10°	10°	10°	10°	10°	10°
Mapping Function	GMF	GMF	GMF	NMF	NMF	NMF

**Table 2:** Summary of IGS stations chosen for the study.

Station	City	Country	Latitude	Longitude	Height
ALGO	Algonquin Park	Canada	45.95861	−78.0714	202
REYK	Reykjavik	Iceland	64.13861	−21.9553	93.1
TIXI	Tixi	Russian Federation	71.63444	128.8664	46.9847
MAL2	Malindi	Kenya	−2.995833	40.1938	−20.4
RIOP	Riobamba	Ecuador	−1.65055	−78.6508	2793.00
NAUR	Nauru	Nauru	−0.55167	166.9253	46.3
PARC	Punta Arenas	Chile	−53.1369	−70.8797	22.3
MAW1	Mawson	Antarctica	−67.6047	62.87056	59.184
MAC1	Macquarie Island	Australia	−54.4994	158.9356	−6.69

**Table 3:** RMSE values in centimeters by groups for day January 27th 2016 and 2017.

	CSRS [cm]	APPS [cm]	MAGIC [cm]	POINT [cm]	RTKLIB [cm]	GLAB [cm]
North 2016	0.48	8.59	0.78	3.90	8.40	4.29
Center 2016	0.98	27.61	1.29	19.06	5.84	4.04
South 2016	0.80	7.39	0.87	20.39	18.67	2.12
North 2017	4.92	7.64	4.96	5.32	10.93	4.12
Center 2017	6.15	25.71	6.18	17.82	6.82	3.07
South 2017	2.55	5.15	2.77	22.36	20.46	2.13

**Table 4:** RMSE values in centimeters by groups for day April 27th 2016 and 2017.

	CSRS [cm]	APPS [cm]	MAGIC [cm]	POINT [cm]	RTKLIB [cm]	GLAB [cm]
North 2016	0.42	7.18	0.80	9.22	10.10	1.6
Center 2016	0.60	31.26	0.86	11.66	14.62	3.99
South 2016	0.86	10.18	0.75	6.45	12.39	2.61
North 2017	0.45	8.96	0.69	3.82	8.19	3.62
Center 2017	0.87	30.64	1.21	12.36	14.09	3.61
South 2017	0.62	4.66	0.69	9.24	13.29	4.76

**Table 5:** RMSE values in centimeters by groups in July 27th 2016 and 2017.

	CSRS [cm]	APPS [cm]	MAGIC [cm]	POINT [cm]	RTKLIB [cm]	GLAB [cm]
North 2016	0.60	14.89	0.98	8.85	5.76	3.17
Center 2016	0.77	20.96	1.22	20.23	11.35	1.77
South 2016	0.68	4.45	0.75	6.97	10.22	2.03
North 2017	0.55	12.36	0.83	12.78	8.59	4.5
Center 2017	0.57	29.70	0.75	16.88	8.71	4.71
South 2017	0.82	8.36	0.77	5.88	8.82	1.38

**Table 6:** RMSE values in centimeters by groups in October 26th 2016 and 2017.

	CSRS [cm]	APPS [cm]	MAGIC [cm]	POINT [cm]	RTKLIB [cm]	GLAB [cm]
North 2016	0.7	21.81	1.07	5.9	3.65	3.6
Center 2016	0.71	18.75	0.8	23.75	16.59	1.77
South 2016	0.7	26.59	0.8	8.59	2.79	4.37
North 2017	0.41	27.93	0.87	7.92	6.54	8.94
Center 2017	0.54	24.05	0.81	18.11	15.36	3.31
South 2017	0.7	41.32	0.8	10.53	2.06	2.73

**Table 7:** RMSE values in centimeters for each software using all data.

	CSRS [cm]	APPS [cm]	MAGIC [cm]	POINT [cm]	RTKLIB [cm]	GLAB [cm]
January 27th 2016	0.78	17.13	1.01	16.23	12.29	3.62
January 27th 2017	4.77	15.67	4.85	16.75	13.96	3.21
April 27th 2016	0.65	19.45	0.80	9.36	12.51	2.91
April 27th 2017	0.67	18.64	0.90	9.45	12.14	4.03
July 27th 2016	0.69	15.06	1.00	13.68	9.42	2.4
July 27th 2017	0.66	17.39	0.78	12.67	8.71	3.85
October 26th 2016	0.70	22.60	0.89	14.97	9.94	3.35
October 27th 2017	0.56	31.99	0.83	12.90	9.71	5.83

## References

[1] Lau L., Cross P. (2007). Investigation into phase multipath mitigation techniques for high precision positioning in difficult environments. J. Navig..

[2] Lau L., Cross P., Steen M. (2012). Flight tests of error-bounded heading and pitch determination with two gps receivers. IEEE Trans. Aerosp. Electron. Syst..

[3] Essen L., Froome D.K. (1951). Dielectric constant and refractive index of air and its principal constituents at 24,000 mc/d. Nature.

[4] Fernandes M.J., Lazaro C., Ablain M., Pires N. (2015). Improved wet path delays for all esa and reference altimetric missions. Remote Sens. Environ..

[5] Awange J.L. (2012). Environmental Monitoring Using GNSS: Global Navigation Satellite Systems.

[6] Bevis M., Chiswell S., Rocken C., van Hove T., Johnson J., Solheim F., Ware R., Businger S. (1994). GPS/STORM-GPS sensing of atmospheric water vapor for meterology. J. Atmos. Ocean. Technol..

[7] Bevis M., Businger S., Chiswell S. (1994). Gps meteorology: Mapping zenith wet delays onto precipitable water. J. Appl. Meteorol..

[8] Hurter F., Maier O. (2014). Tropospheric profiles of wet refractivity and humidity from the combination of remote sensing data sets and measurements on the ground. Atmos. Meas. Tech..

[9] Nilsson T., Elgered G. (2008). Long-term trends in the atmospheric water vapor content estimated from ground-base GPS data. J. Geophys. Res..

[10] Rohm W., Yuan Y., Bertukan B., Zhang K., Le Marshall J. (2014). Ground-based gnss ztd/iwv estimation system for numerical weather prediction in challenging weather conditions. Atmos. Res..

[11] Dong Z., Jin S. (2018). 3-D water vapor tomography in Wuhan from GPS, BDS and GLONASS observations. Remote Sens..

[12] Heroux P., Kouba J. (2001). GPS precise point positioning using IGS orbit products. Phys. Chem. Earth.

[13] Urquhart L., Santos M.C., Garcia C.A., Langley R., Leandro R.F. (2014). Global assessment of UNB’s online precise point positioning software. International Association of Geodesy Symposia.

[14] Leandro R.F., Santos M.C., Langley R.G. (2011). Analyzing GNSS data in precise point positioning software. GPS Solut..

[15] Dawidowicz K., Krzan G. (2014). Coordinate estimation accuracy of static precise point positioning using on-line PPP service, a case study. Acta Geod. Geophys..

[16] Mendonca M., White R.M., Santos M.C., Langley R.B. (2016). Assessing GPS + Galileo precise point positioning capability for integrated water vapor estimation. International Association of Geodesy Symposia.

[17] Guo Q. (2015). Precision comparison and analysis of four online free PPP services in station positioning and tropospheric delay estimation. GPS Solut..

[18] Ahmed F., Vaclavovic P., Teferle F.N., Dousa J., Bingley R., Laurichesse D. (2014). Comparative analysis of real-time precise point positioning zenith total delay estimates. GPS Solut..

[19] Hofmann-Wellenhof B., LIchtenegger H., Wasle E. (2008). GNSS-Global Navigation Satellite Systems GPS, Glonass, Galileo and More.

[20] Wilgan K., Hurter F., Geiger A., Rohm W., Bosy J. (2016). Tropospheric refractivity and zenith path delays from least-squares collocation of meteorological and GNSS data. J. Geod..

[21] Gao Y., Chen K. (2005). Performance analysis of precise point positioning using real-time orbit and clock products. J. Glob. Position. Syst..

[22] Zumberge J.F., Heflin M.B., Jefferson D.C., Watkins M.M., Webb F.H. (1997). Precise point positioning for the efficient and robust analysis of GPS data from large networks. J. Geophys. Res..

[23] Gao Y. (2006). GNSS solutions: Precise point positioning and its challenges. InsideGNSS.

[24] JPL Automatic Precise Point Positioning Service. http://apps.gdgps.net/.

[25] Sanz J., Rovira-Garcia A., Hernandez M., Juan J., Ventura-Traveset J., Lopez C., Hein G. The ESA/UPC GNSS-Lab tool (gLAB): An advanced educational and professional package for GNSS data processing and analysis. Proceedings of the 6th ESA Workshop on Satellite Navigation Technologies Multi-GNSS Navigation Technologies.

[26] Sanz Subirana J., Juan Zornoza J.M., Hernandez-Pajares M. (2013). GNSS Data Processing: Volume I, Fundamentals and Algorithms.

[27] Sanz Subirana J., Juan Zornoza J.M., Hernandez-Pajares M. (2013). GNSS Data Processing Volume II: Laboratory Exercises.

[28] Mohammed J., Moore T., Hill C., Bingley R.M., Hansen D.N. (2016). An assessment of static precise point positioning using GPS only, GLONASS only and GPS plus GLONASS. Measurement.

[29] IGS Igs Stations. http://www.igs.org/network/.

